# Field evaluation of quantitative point of care diagnostics to measure glucose-6-phosphate dehydrogenase activity

**DOI:** 10.1371/journal.pone.0206331

**Published:** 2018-11-02

**Authors:** Mohammad Shafiul Alam, Mohammad Golam Kibria, Nusrat Jahan, Kamala Thriemer, Mohammad Sharif Hossain, Nicholas M. Douglas, Ching Swe Phru, Wasif Ali Khan, Ric N. Price, Benedikt Ley

**Affiliations:** 1 Infectious Diseases Division, International Centre for Diarrheal Diseases Research, Bangladesh, Mohakhali, Dhaka, Bangladesh; 2 Global and Tropical Health Division, Menzies School of Health Research and Charles Darwin University, Darwin, Australia; 3 Centre for Epidemiology and Biostatistics, Melbourne School of Population and Global Health, The University of Melbourne, Melbourne, Australia; 4 Centre for Tropical Medicine and Global Health, Nuffield Department of Clinical Medicine, University of Oxford, Oxford, United Kingdom; Instituto Rene Rachou, BRAZIL

## Abstract

**Background:**

Glucose-6-Phosphate dehydrogenase (G6PD) deficiency is the most common enzymopathy worldwide, no reliable bedside diagnostic tests to quantify G6PD activity exist. This study evaluated two novel quantitative G6PD diagnostics.

**Methods:**

Participants with known G6PD activity were enrolled in Bangladesh. G6PD activity was measured by spectrophotometry, Biosensor (BS; AccessBio/CareStart, USA) and STANDARD G6PD (SG; SDBiosensor, ROK). G6PD activity was measured repeatedly in a subset of samples stored at room temperature and 4°C.

**Results:**

158 participants were enrolled, 152 samples tested by BS, 108 samples by SG and 102 samples were tested by all three methods. In comparison to spectrophotometry BS had sensitivity and specificity of 72% (95%CI: 53–86) and 100% (95%CI: 97–100) at 30% cut off respectively, while SG had a sensitivity of 100% (95%CI: 88–100) and specificity of 97% (95%CI: 91–99) at the same cut off. The sensitivity and specificity at 70% cut off activity were 71% (95%CI: 59–82) and 98% (95%CI, 92–100) respectively for BS and 89% (95%CI: 77–96) and 93% (95%CI: 83–98) respectively for SG. When an optimal cut-off was applied the sensitivity of the BS at 70 cut off rose to 91% [95%CI: 80–96] and specificity to 82% [95%CI: 83–89]; a diagnostic accuracy comparable to that of the SG (p = 0.879). G6PD activity dropped significantly (-0.31U/gHb, 95%CI: -0.61 to -0.01, p = 0.022) within 24 hours in samples stored at room temperature, but did not fall below 90% of baseline activity until day 13 (-0.87U/gHb, 95%CI: (-1.11 to -0.62), p<0.001).

**Conclusion:**

BS and SG are the first quantitative diagnostics to measure G6PD activity reliably at the bedside and represent suitable alternatives to spectrophotometry in resource poor settings. If samples are stored at 4°C, G6PD activity can be measured reliably for at least 7 days after sample collection.

## Introduction

Glucose-6-Phosphate dehydrogenase deficiency (G6PDd) is the most common inherited enzymopathy worldwide, caused by genetic polymorphisms of the gene located on the X chromosome. The G6PD enzyme is the rate limiting step of the pentose-phosphate-pathway (PPP), responsible for reducing nicotinamide adenine dinucleotide phosphate (NADP^+^) to NADPH. The latter maintains sufficiently high concentrations of glutathione, essential for binding radicals and protecting human cells from the adverse effects of oxidative stress [1]. Red blood cells (RBCs) are particularly vulnerable to oxidative stress and, in the absence of a nucleus, are reliant upon the G6PD generated during erythropoiesis. RBCs with reduced G6PD activity are more vulnerable to oxidative stress and have a significantly shorter half-life [1].

G6PDd affects an estimated 400 million individuals [2], with more than 185 clinically relevant variants reported to date [3]. While G6PDd does not affect quality of life or life expectancy in the great majority of affected individuals, it is a significant risk factor for hyperbilirubinemia and kernicterus in newborns [4] and can result in haemolysis induced by a wide range of compounds including fava beans, sulphonamides, quinolones, dapsone and the 8-aminoquinoline class of antimalarials [1]. The degree of haemolysis associated with G6PDd depends upon the specific genetic variant, the age of the RBC population and the nature of the exposure to the trigger factor.

G6PDd offers protection against severe falciparum malaria and, in areas of intense malaria transmission, this provides selective pressure resulting in a high prevalence of G6PDd [5–7]. Primaquine, an 8-aminoquinoline compound, is the only widely available antimalarial agent with activity against the late gametocyte stages of *Plasmodium falciparum* (*P*. *falciparum*) and the hypnozoite stages of *P*. *vivax*. It is an essential drug for achieving radical cure of malaria. However its clinical use raises a public health dilemma since the benefits of radical cure need to be weighed against the dangers of severe haemolysis in vulnerable individuals with G6PD deficiency [8]. Several countries have introduced routine screening for G6PDd in neonates [9] and the current WHO malaria treatment guidelines recommend that where possible, G6PD testing should be undertaken prior to administration of primaquine for radical cure[10].

A reliable diagnosis of G6PD status by spectrophotometry is costly, has a turn-around time of several hours, and requires good laboratory infrastructure; limitations that render routine G6PD testing by spectrophotometry unsuitable in most malaria endemic countries [11]. The majority of field applications suitable for the diagnosis of G6PDd provide a qualitative result, reliably identifying only individuals with less than 30% enzyme activity [11]. Since G6PDd is an X-linked disorder, females can be fully deficient (homozygotes), partially deficient (heterozygotes) or G6PD normal, whereas males are either hemizygote deficient or normal. The degree of deficiency in heterozygous females varies due to random X-inactivation (lyonisation) [12]. Current qualitive tests are unsuitable for females heterozygous for the G6PD gene who may have a G6PD normal qualitative result whilst still being at risk of drug induced haemolysis [13]. Heterozygous females can only be diagnosed by either quantitative assays, flow-cytometry or genetic testing. Tafenoquine is a new 8-aminoquinoline under development which can achieve radical cure with a single dose [14]. However, in view of its sustained activity in the blood it is expected to be contraindicated in females with an enzyme activity below 70%. The diagnosis of patients with intermediate G6PD activity will require novel quantitative point of care G6PD diagnostics, capable of determining haemolytic risk in all individuals.

The aim of the current study was to evaluate two quantitative point of care (PoC) assays (biosensors) and compare them with spectrophotometry. In addition, the stability of the G6PD enzyme activity was studied under different storage conditions and with different time intervals between blood collection and reference testing.

## Methods

*Study procedures*: Participants were recruited in the Chittagong Hill Tracts, Bangladesh from a cohort of patients with known G6PD status determined from previous studies [15, 16]. Individuals were selected by convenience sampling to ensure testing samples with a broad range of G6PD activities.

After obtaining written informed consent, a total of 5ml of venous blood was collected by a single use syringe and the sample tested immediately for G6PD activity and hemoglobin (Hb) concentration using the STANDARD G6PD TEST (SG) manufactured by SDBiosensor (Suwon-si, Republic of Korea). The remaining blood was inserted into an EDTA vacutainer, stored at 4°C and transported within 24 hours to the capital Dhaka, 390 km from the field site. Upon arrival all samples were retested for G6PD activity using three methods: i) the SG, ii) the Biosensor (BS, manufactured by Accessbio, Somerset, USA) and iii) spectrophotometry using a Shimadzu UV-1800 (Shimadzu, Kyoto, Japan). All G6PD measurements were undertaken in duplicate and if there was an error message, a third measure was taken. G6PD activities were subsequently normalized for hemoglobin (Hb). The SG provides a measurement of G6PD activity and Hb in the same sample and during the same measurement, G6PD activities were normalized accordingly. Accessbio/Carestart provides a Hb reading device (MHD-1) complimentary to the BS which was used to manually normalize the BS readings, while the results from the reference method spectrophotometry were normalized by results from a complete blood count (CBC) performed at the reference using a XN-1000 (Sysmex Corporation, Kobe, Japan).

A subset of 50 samples was divided into two aliquots, one of which was stored at room temperature (approx. 24–26°C) and the other kept at 4°C. G6PD activity was retested by spectrophotometry in samples stored at room temperature 1, 2, 4, 6, and 24 hours after the first measurement and in refrigerated aliquots on 3, 6, 9, 13 and 20 days after the first measurement.

### Assay procedures

As part of quality assurance processes the BS (Cat. No: BGB-E00182) was assessed daily using the control strip provided by the manufacturer. Participant samples were only processed once the functionality of the Biosensor had been confirmed. For each sample, the test strip was dipped into venous blood and 5μl of blood pulled into the test chamber by capillary force. The machine produced an uncorrected measure of enzyme activity (U/dL) within 4 minutes and this was normalized using a single measure of the Hb concentration, quantified with the Carestart MHD-1 machine.

For the SG Test device, 10μl of venous blood were added to the test buffer with a single use pipette and mixed. 10μl of the blood-buffer solution was then added to the test chamber of the test strip. The device reported G6PD activity as U/gHb as well as a separate Hb measurement within two minutes.

G6PD spectrophotometry was performed at 37°C testing temperature, using G6PD–G7583 kits (Pointe Scientific (PS), Canton, USA) and analyzed on a Shimadzu 1800 spectrophotometer (Kyoto, Japan). All spectrophotometry measurements were run in duplicate, and if values differed by more than 10% of the upper value, a third measure was taken and only the two closest measures were considered. G6PD deficient (Cat. No.: HC-108DE), intermediate (Cat. No.: HC-108IN) and normal controls (Cat. No.: HCS-108) (all from ACS Inc., Fishers, USA) were run every day prior to sample testing. The enzyme activity for spectrometry was normalized using the Hb derived from the CBC.

### Statistical analysis

Data were entered in Epidata version 3.1 (Denmark) and analysed using Stata version 14 (Stata Corp, USA). G6PD measurements recorded by the BS and SG were compared with the reference method spectrophotometry using Pearson correlation (r) or Spearman’s rank correlation (r_s_) as appropriate and Bland-Altman plots. The Hb results derived from MHD-1 and SG were assessed similarly, considering CBC as reference. The difference between matched pairs was assessed using the signed ranks Wilcoxon test. Correlation coefficients were compared using Fisher r-to-z transformation.

A total of 47 males and females had been identified by spectrophotometry as G6PD normal in previous studies [15, 16]. Spectrophotometry results of these individuals collected in the course of this study were used to define the median enzyme activity according to SG, BS and spectrophotometry, defined as 100% G6PD activity. G6PD activity of all individuals was then calculated as a percentage of the median value for each test. All G6PD results were categorized according to the current threshold used to determine exclusion from primaquine treatment (<30% activity) [17] and tafenoquine (<70% activity). In a supplementary analysis data were also categorized above and below 60% activity according to the WHO definition of intermediate activity [18]. Categorical data were compared using Chi-square test, Fishers exact test and McNemar’s test for correlated proportions as appropriate. Sensitivity and specificity were calculated for different threshold activities applying standard formulas [19, 20].

The area under the Receiver Operating Curve (ROC) was calculated and the areas under the curve (AUC) compared for each assay applying 30% and 70% cut-off activity, assuming spectrophotometry as the reference method. Assay specific optimal cut–offs were calculated prioritizing assay sensitivity. If a cut-off could not be determined to produce sensitivity and specificity above 95%, then a value was selected to generate the highest sensitivity for a specificity greater than 80%.

The decay in G6PD activity over time, quantified by spectrophotometry, was assessed by paired t-test and a mixed-effects linear regression model was used to predict the time at which G6PD activity fell below 90% of the initial value.

### Ethics

The study was approved by the ethical review committee (ERC) and research review committee (RRC) of the icddr,b (PR-17043) and the Australian Human Research Ethics Committee (HREC) of the Northern Territory (HREC 17.2771). Prior to enrolment all participants provided written informed consent to participate and publication of results.

## Results

Between 10^th^ September and 20^th^ December 2017, a total of 158 individuals were enrolled in the study (S1 Data). Participants were predominantly female (115, 73%), with a mean Hb concentration of 12.9 g/dL (95CI: 12.6 to 13.1) by CBC. The median normal G6PD activity defined in 47 previously identified G6PD normal individuals was 9.9 U/gHb (range: 7.5–16.2) and this value was taken to define 100% G6PD activity. Six (4%) participants had less than 10% G6PD activity, 31 (20%) had between 10% and 30%, 32 (20%) had activities between 30% and 70% and 89 (57%) participants had G6PD activities greater than 70%.

### Laboratory assessment

The mean delay between sample collection and processing in the reference laboratory was 23.3 hours (range 20.8 to 26.5h). At the reference laboratory, G6PD status was assessed in all 158 participants by spectrophotometry, 152 (96%) participants by BS and 108 (68%) by SG; 102 participants (65%) were tested by all three methods. The low number of samples processed using SG was attributable to the device only becoming available 4 weeks after the start of enrolment. In 6 participants BS wasn’t attempted. G6PD activity by spectrophotometry did not correlate with the delay between sampling and processing (r = -0.011, p = 0.890) (S1 Fig).

**Fig 1:**
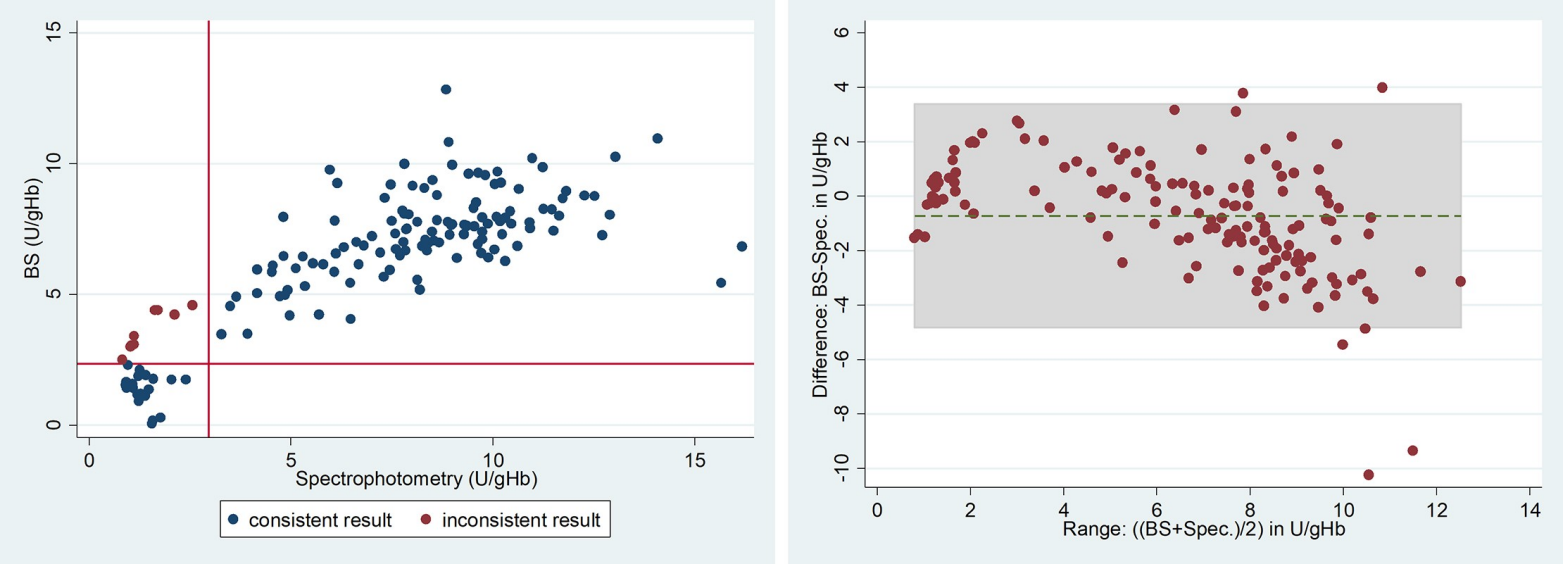
**Comparison of the Biosensor (BS) with spectrophotometry (Spec) a) Scatter plot and b) Bland-Altman plot.** Inconsistent results: results with <30% by one method, but not the other; a) r_s_ = 0.7729; p<0.001, n = 152, red lines indicate 30% cut off. b) Mean difference: 0.72 U/gHb, 95% LoA: -4.82 to 3.38 U/gHb (grey shaded area).

**Fig 2:**
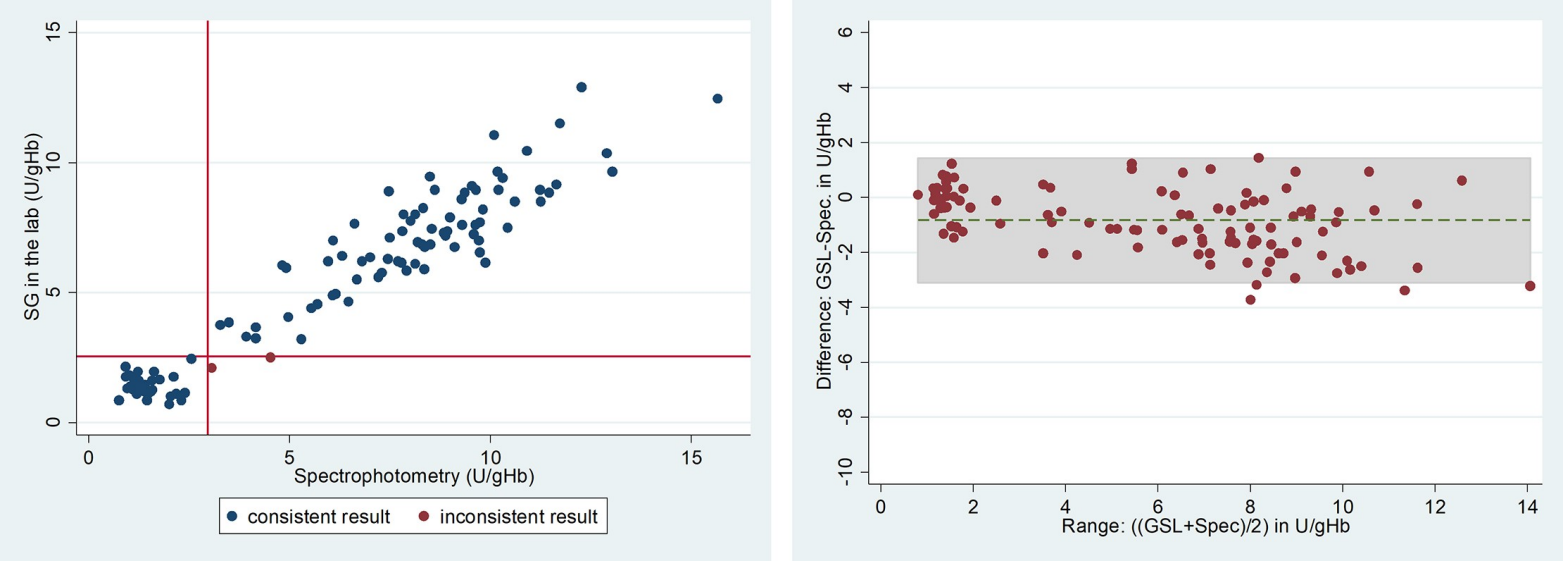
**Comparison of the STANDARD G6PD TEST with spectrophotometry a) Scatter plot and b) Bland-Altman Plot.** Inconsistent results: results with <30% by one method, but not the other; a) r_s_ = 0.9145; p<0.001, n = 108, red lines indicate 30% cut-off. b) Mean difference: 0.83 U/gHb, 95% LoA: -3.10 to 1.44 U/gHb (grey shaded area).

The correlation coefficient (r_s_) for activity measurements from the BS and spectrophotometry was 0.773 (p<0.001), with a mean difference of -0.72 U/gHb (Range: -10.20 to 4.00) (Fig 1) and for the SG and spectrophotometry was r_s_ = 0.915 (p<0.001) with a mean difference of -0.83 U/gHb (range -3.70 to 1.40)(Fig 2).

**Fig 3:**
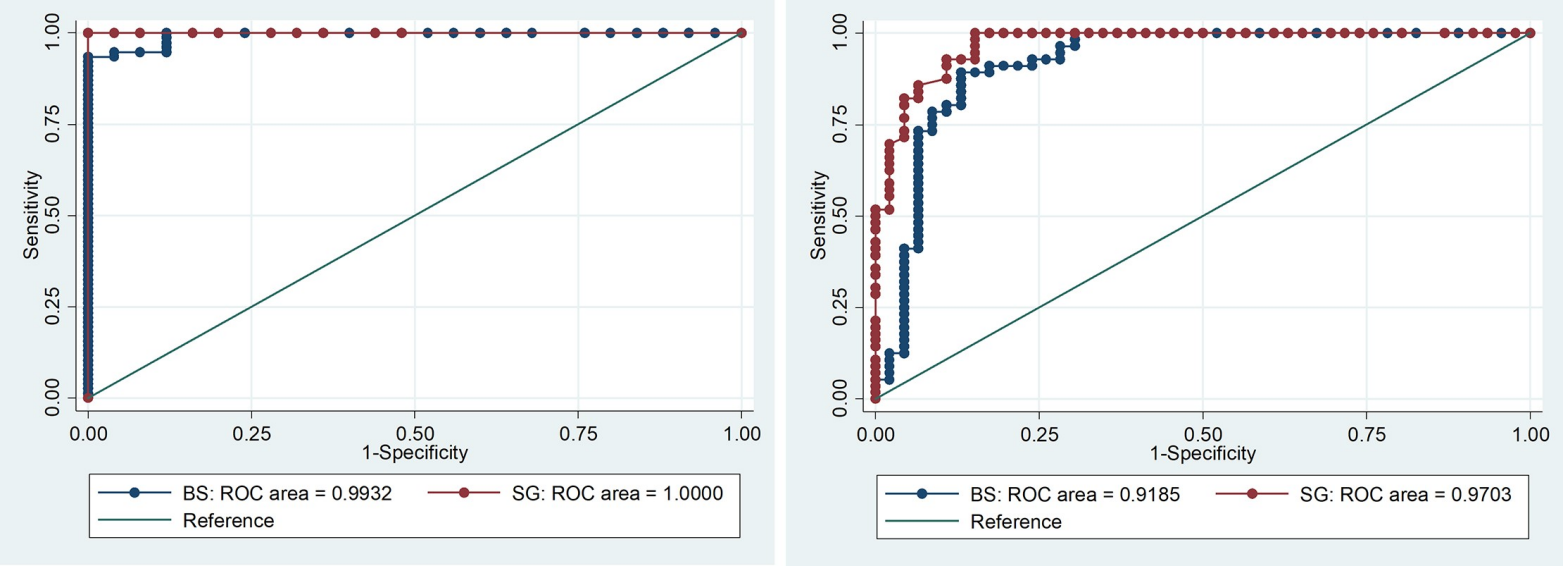
**Receiver operator curves for the two biosensors at the 30% (a) and 70% (b) threshold activities done in the laboratory.** a) p = 0.165, n = 102 b) p = 0.068, n = 102.

At the 30% activity threshold the area under the receiver operator curve (ROC) was 0.994 (95%CI 0.987–1.000) for BS and 0.999 (95%CI 0.997–1.000) for SG. In the 102 samples in which all three measurements were recorded, the areas under the curve did not differ significantly; p = 0.130 (Fig 3). The sensitivity and specificity for BS at the 30% threshold were 72% (95%CI: 53–86) and 100% (95%CI: 97–100) respectively. For SG the corresponding values were 100% (95%CI: 88–100) and 97% (95%CI: 91–99). At this threshold, the optimal cut-off for BS was 4.6 U/gHb (59% assays specific G6PD activity), which classified 95% (145/152) of samples correctly (sensitivity: 97% [95%CI: 84–100], specificity: 95% [95%CI: 89–98]). The corresponding optimal cut off for SG was 2.5 U/gHb (29% assay specific G6PD activity), classifying 99% (107/108) of all samples correctly (sensitivity: 100% [95%CI: 88–100], specificity: 99% [95%CI: 93–100]) (Table 1).

**Table 1:** Performance in the reference laboratory and optimal cut off of Biosensor and STANDARD G6PD TEST at different thresholds.

Cut—off	Test	Sensitivity (95%CI)	Specificity (95%CI)	Optimal cut off in U/gHb (% of assay specific activity)	Sensitivity at optimal cut-off	Specificity at optimal cut off
**30%**	**Biosensor**	72% (53–86) (23/32)	100% (97–100) (120/120)	4.6 (59)	97% (84–100) (31/32)	95% (89–98) (114/120)
	**STANDARD G6PD TEST**	100% (88–100) (30/30)	97% (91–100) (76/78)	2.5 (29)	100% (88–100) (30/30)	99% (93–100) (77/78)
**60%**	**Biosensor**	71% (57–83) (37/52)	99% (95–100) (99/100)	6.7 (86)	98% (90–100) (51/52)	81% (72–88) (81/100)
	**STANDARD G6PD TEST**	95% (84–99) (41/43)	95% (87–99) (62/65)	4.6 (53)	93% (81–99) (40/43)	100% (95–100) (65/65)
**70%**	**Biosensor**	71% (59–82) (45/63)	98% (92–100) (87/89)	6.8 (87)	91% (80–96) (57/63)	82% (73–89) (73/89)
	**STANDARD G6PD TEST**	89% (77–96) (46/52)	93% (83–98) (52/56)	6.4 (75)	94% (84–99) (49/52)	82% (70–91) (46/56)
**80%**	**Biosensor**	69% (57–79) (55/80)	96% (88–99) (69/72)	6.9 (89)	80% (70–88) (64/80)	82% (71–90) (59/72)
	**STANDARD G6PD TEST**	90% (80–96) (56/62)	85% (71–94) (39/46)	6.6 (77)	90% (80–96) (56/62)	91% (79–98) (42/46)

TP: True positive, TN: True negative, FP: False positive, FN: False negative. Positive = G6PD deficient, Negative = G6PD normal. Sensitivity defined as TP/(TP+FN) and specificity defined at TN/(TN+FN)

At the 70% threshold the area under the curve for BS was 0.940 (95%CI: 0.895–0.984) and for SG was 0.974 (95%CI: 0.949–0.999). In samples which were processed by all three assays, there was no significant difference in the area under the curves for SG and BS (p = 0.170) (Fig 3). The sensitivity of BS for detecting individuals with an enzyme activity less than 70% was 71% (95%CI: 59–82) and the specificity 98% (95%CI: 92–100). The corresponding values for SG were 89% (95%CI: 77–96) and 93% (95%CI: 83–98) respectively. The optimal cut-off for defining the 70% threshold was 6.8 U/gHb for BS (87% assay specific activity), correctly classifying 86% (130/152) of samples (sensitivity: 91% [95%CI: 80–96], specificity: 82% [95%CI: 73–89]) whereas the corresponding value for SG was 6.4 U/gHb (75% assay specific activity), which classified 88% (95/108) of samples correctly (sensitivity: 94% [95%CI: 84–99], specificity: 82% [95%CI: 70–91]) (Table 1).The data for each assay to define the WHO threshold for intermediate activity (60%) and 80% are presented in Table 1.

### Assessment in the field (SG only)

Only the SG device was assessed in the field. In the 106 (67%) samples assessed, the first and second measurements taken immediately after one another were closely correlated (r_s_ = 0.903, p<0.001) with a mean difference of -0.66 U/gHb (range -6.30 to 2.50) (Table 2 and S2 Fig). A 3^rd^ measurement was therefore not required for any of the samples. The area under the curve was significantly greater at 30% cut off (1.000, 95CI:0.999–1.000) than at 70% cut off activity (0.926, 95%CI: 0.875–0.977), p = 0.014.

**Table 2:** Performance and optimal cut-off of SG at different threshold activities in the field.

Cut–off	Sensitivity in % (95%CI)	Specificity in %(95%CI)	Optimal cut off in U/gHb (% of assay specific activity)	Sensitivity at optimal cut-off in %	Specificity at optimal cut off in %
**30%**	100% (88–100) (30/30)	97% (91–100) (74/76)	2.1 (27)	100% (88–100) (30/30)	100 (95–100) (76/76)
**60%**	93% (81–99) (40/43)	94% (85–98) (59/63)	5.2 (67)	95% (84–99) (41/43)	84% (73–92) (53/63)
**70%**	90% (79–97) (46/51)	87% (76–95) (48/55)	5.7 (74)	94 (84–99) (48/51)	86% (73–94) (47/55)
**80%**	92% (82–97) (56/61)	80% (64–90) (36/45)	6.3 (82)	95% (86–99) (58/61)	80% (65–90) (36/45)

TP: True positive, TN: True negative, FP: False positive, FN: False negative. Positive = G6PD deficient, Negative = G6PD normal. Sensitivity defined as TP/(TP+FN) and specificity defined at TN/(TN+FN)

SG measures taken in the field correlated well with measures taken at the reference laboratory (r_s_ = 0.877, p<0.001) with a mean difference of 0.33 U/gHb (range: -4.700 to 7.550). The SG measurements in the field also correlated well with spectrophotometry (r_s_ = 0.912, p<0.001) with a mean difference of -1.18 U/gHb (range: - 5.22 to 5.45) (S3 and S4 Figs).

The number of samples classified above and below the 30% and 70% thresholds did not differ significantly between the field and reference laboratory SG measurements (p = 1.000 and p = 0.456 respectively), nor between the SG field measurement and spectrophotometry (both p = 1.000). When categorizing results at the 30% threshold by spectrophotometry, SG in the field had 100% sensitivity (95%CI: 88–100) and 97% (95%CI: 91–100) specificity. At the 70% threshold, the sensitivity of the SG in the field fell to 90% (95%CI: 79–97) and specificity to 87% (95%CI:76–95). The optimal cut off for SG at 30% enzyme activity was 2.1U/gHb identifying all samples correctly. At 70% enzyme activity the optimal cut off was 5.7U/gHb, resulting in a sensitivity of 94% [95%CI: 84–99] and specificity of 86% [95%CI: 73–94]; Table 2.

### Performance of the MHD-1 and STANDARD G6PD Test measuring Hb concentration

At the reference laboratory, the Hb concentration was measured by CBC in 158 samples with a mean concentration of 12.9 g/dL (95%CI 12.6–13.1).

The Hb concentrations from the MHD-1 device correlated closely with that from the CBC (r_s_ = 0.861, p<0.001), with a mean difference of -0.48 g/dL (range: -3.50 to 2.60), corresponding to a variation of 4% of the mean Hb value measured by MHD-1 (S5 Fig). The correlation of Hb concentration derived by SG and the CBC was r_s_ = 0.890 (p<0.001, n = 108) with a mean difference of 0.4 g/dL (range: -2.8 to 1.6), corresponding to a variation of 3%; (S5 and S6 Figs). When comparing G6PD activities of BS normalized by the Hb measurement from MHD to the same readings normalized by the Hb measurements from the CBC, the mean difference was -0.23 U/gHb (95% Limits of agreement (95%LoA): -1.05 to 0.58), when comparing G6PD activities measured by the SG and normalized by its internal Hb measurement against the same reading normalized by the Hb reading from the CBC the mean difference was 0.14 U/gHb (95% Limits of agreement (95%LoA): -0.04 to 0.06).

### Variation of G6PD activity over time

**Fig 4:**
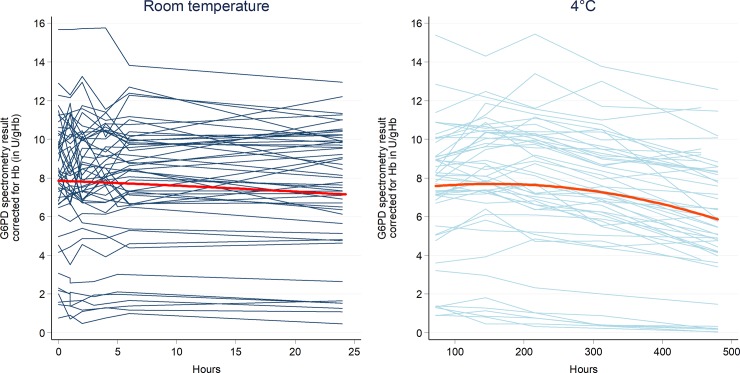
Absolute drop in G6PD activity over time under different storage conditions.

A total of 50 samples were assessed repeatedly by spectrophotometry after intervals of storage at room temperature and 4°C. The temperature of the samples stored at room temperature ranged from 24°C to 26°C. In this environment the mean G6PD activity fell from 7.9 U/gHb (95%CI: 7.0–8.8) at baseline to 7.6 U/gHb (95%CI: 6.7–8.5) at 24 hours; a mean fractional fall of 5.4% (p = 0.022). When stored at 4°C the overall G6PD activity fell from a mean of 7.9 U/gHb (95%CI: 7.0 to 8.8) to 7.5 U/gHb (95%CI: 6.6 to 8.4) within 3 days (p = 0.001), a fractional fall of 6%. A fall of greater than 10% was not observed at room temperature within the first 24 hours, but at 4°C was observed after 13 days (Table 3 and S7 Fig). In a mixed effect model, predicted G6PD activity levels remained within 10% of the initial value until 13 days after the first measurement for samples stored at 4 degrees Celsius (Fig 4). No significant correlation between G6PD activity at baseline and drop in G6PD activity within 24 hours and within 13 days was observed (p = 0.100 and, p = 0.883).

**Table 3:** Change in G6PD activity between the first measurement by spectrophotometry in the laboratory and after storage at room temperature and 4°C.

Mean delay between time 0 and test point (range)	Storage temperature	Mean absolute change in G6PD activity compared to baseline in U/gHb (95%CI) *^#^	Mean fractional fall in enzyme activity in % (95%CI)^#^	P value^#^
1.2 hours (1.0–1.4)	*Room temperature*	-0.10 (-0.38 to 0.18)	-2.3 (-6.8 to 2.2)	0.241
2.1 hours (1.9–2.4)	*Room temperature*	-0.17 (-0.44 to 0.11)	-3.5 (-7.5 to 0.4)	0.112
4.1 hours (3.8–4.6)	*Room temperature*	-0.18 (-0.44 to 0.08)	-3.0 (-6.4 to 0.5)	0.080
6.1 hours (5.8–6.6)	*Room temperature*	-0.15 (-0.39 to 0.09)	-1.3 (-5.0 to 2.3)	0.109
24.3 hours (23.9–25.7)	*Room temperature*	-0.31 (-0.61 to -0.01)	-5.4 (-9.7 to -1.0)	**0.022**
3.0 days (3.0–3.0)	*4°C*	-0.43 (-0.70 to -0.17)	-6.4 (-10.6 to -2.2)	**0.001**
6.0 days (6.0–6.0)	*4°C*	-0.05 (-0.29 to 0.20)	-2.5 (-7.7 to 2.7)	0.356
9.0 days (9.0–9.0)	*4°C*	-0.26 (-0.51 to 0.01)	-8.2 (-14.2 to -2.3)	**0.025**
13.0 days (13.0–13.0)	*4°C*	-0.87 (-1.11 to -0.62)	-17.2 (-23.9 to -10.5)	**<0.001**
19.8 days (19.0–20.0)	*4°C*	-1.9 (-2.2 to -1.6)	-31.3 (-38.7 to -23.8)	**<0.001**

*At baseline the mean G6PD activity was 7.92 U/gHb

^#^compared to baseline

## Discussion

We present data from two novel devices to quantify G6PD activity at the point of care. Each device was compared with spectrophotometry using kits from Pointe Scientific and both devices showed high correlation [21] with the reference method spectrophotometry.

The performance of the SG test was assessed under both field and reference laboratory conditions. In the laboratory SG showed an almost perfect correlation with spectrophotometry at 30% cut-off activity, which represents the threshold currently applied to guide hypnozoitocidal primaquine treatment [18]. Areas under the curve for BS and SG at 30% cut-off did not differ significantly, suggesting that the BS has a comparable discriminatory power. Whilst these results are reassuring, a number of qualitive G6PD diagnostics already available are capable of diagnosing G6PD deficiency at the same threshold with comparable accuracy at lower costs [11]. The main clinical use for quantitative point of care diagnostics is to provide a more accessible way of defining G6PD activity at the bedside and to identify patients with intermediate G6PD activity below 70% required for identifying heterozygous females and those in whom tafenoquine should not be prescribed. At this threshold all current qualitative tests perform poorly [8, 14]. The SG performed well at this threshold with a sensitivity of almost 90% at 70% cut off activity, while the sensitivity of BS was 70%. When an optimal cut-off for the BS was applied the sensitivity and specificity at 70% cut off were similar to that of the SG, however the clinically relevant window between 30% and 70% G6PD activity was narrow, ranging from 4.6 U/gHb to 6.8 U/gHb, whereas the corresponding window for SG ranged from 2.5 U/gHb to 6.4 U/gHb, suggesting a greater granularity of SG (Table 1, Fig 3).

The SG was also evaluated under field conditions and showed a consistent performance as to that in the laboratory. No significant differences in sensitivity and specificity at 70% and 80% cut-off activity between field and laboratory results were found. Furthermore, there was excellent repeatability of field SG results. Indeed, a single measurement in the field reliably identified 9 out of 10 individuals with an enzyme activity less than 70%.

The BS determines G6PD activity by measuring the electrochemical properties of a blood sample whereas the SG and spectrophotometry determine G6PD activity through a colorimetric reaction. The BS device was simple to use, with the sample collected directly from a fingerpick and dragged into the measuring chamber by capillary forces. In contrast the SG requires a specified amount of blood to be pipetted to a buffer and the blood buffer solution subsequently needs to be transferred with a pipette to the test field of the machine. The BS device costs approximately 670 USD, with each strip costing 3.40 USD, while the SG device costs approximately 380 USD with single use strips costing 3 USD per test. While the BS requires a separate Hb measurement and results must be normalized manually, the SG has an integrated Hb measurement, providing normalized G6PD activity and a Hb measurement from the same sample and strip.

Both methods for measuring Hb performed well against the reference method (CBC), with a mean difference of less than 0.5 g/dL, an acceptable degree of accuracy for most clinical applications and thus suitable alternatives to other PoC Hb measurement devices. Comparable findings for the MHD-1 had been reported earlier, underlining its consistent performance [15].

Our study addressed the practical application of G6PD testing and the duration for which samples could be stored before testing. At room temperature G6PD activity derived from spectrophotometry remained stable initially but had decreased by 5% at 24 hours. The drop in G6PD activity appeared to be independent of the underlying G6PD activity, suggesting that the absolute decay is not G6PD variant specific. At 4°C, G6PD activity did not fall by more than 10% until after 9 days of storage. Hence the current recommendation that samples should be stored at 4°C and processed within 7 days after sample collection is conservative but valid.

In conclusion, significant progress has been made to develop point of care quantitative measurement of G6PD activity. Our study presents field data gathered for two novel diagnostics, both of which performed well under field and laboratory conditions with comparable accuracy. These tests offer, for the first time, reliable alternatives to spectrophotometry in settings with poor laboratory infrastructure, and have potential to facilitate the safe roll out of the radical cure of *P*. *vivax* malaria.

## Supporting information

S1 Fig G6PD activity (U/gHb) and the delay from sample collection and processing. r = 0.0030; p = 0.970, n = 158.
(PDF)

S2 Fig **Repeatability of two measurements of the STANDARD G6PD TEST in the field- a) Scatter plot and b) Bland–Altman plot**.
a) rs = 0.9025; p<0.001, n = 106 b) Mean difference: - 0.66 U/gHb, 95% LoA: -2.04 to 3.36 U/gHb (grey shaded area).
(PDF)

S3 Fig **Comparison of the STANDARD G6PD Test (SG) in the lab and field a) Scatter plot and b) Bland-Altman plot**.
a) rs = 0.8765; p<0.001, n = 106 b) Mean difference: -0.33 U/gHb, 95% LoA: -3.29 to 2.63 U/gHb (grey shaded area).
(PDF)

S4 Fig **Comparison of the STANDARD G6PD Test (SG) in the field against spectrophotometry a) Scatter plot and b) Bland-Altman plot**.
a) rs = 0.9122; p<0.001, n = 106 b) Mean difference: -1.18 U/gHb, 95% LoA: -4.20 to 1.84 U/gHb (grey shaded area).
(PDF)

S5 Fig **Comparison of the MHD-1 in the lab against CBC-HB a) Scatter plot and b) Bland-Altman plot**.
a) rs = 0.8614; p<0.001, n = 158 b) Mean difference: 0.48 g/dL, 95% LoA: -1.98 to 1.01 g/dL (grey shaded area).
(PDF)

S6 Fig **Comparison of the SG-Hb in the lab against CBC-HB a) Scatter plot and b) Bland-Altman plot**.
a) rs = 0.8892; p<0.001, n = 108 b) Mean difference: 0.37 g/dL, 95% LoA: -0.80 to 1.54 g/dL (grey shaded area).
(PDF)

S7 Fig Absolute fall in G6PD activity at 24 hrs and 13 days. Grey shaded area indicates 10% of measurement, equivalent to max. variation of spectrophotometry.
(PDF)

S1 Data Corresponding database. (XLSX)
