# Supplementary figures and images for: Field evaluation of quantitative point of care diagnostics to measure glucose-6-phosphate dehydrogenase activity
Source: PLoS One. 2018 Nov 2;13(11):e0206331. doi: 10.1371/journal.pone.0206331 (PMC6214512; doi:10.1371/journal.pone.0206331)

**Supp. Figure 1: G6PD activity (U/gHb) and the delay from sample collection and processing**

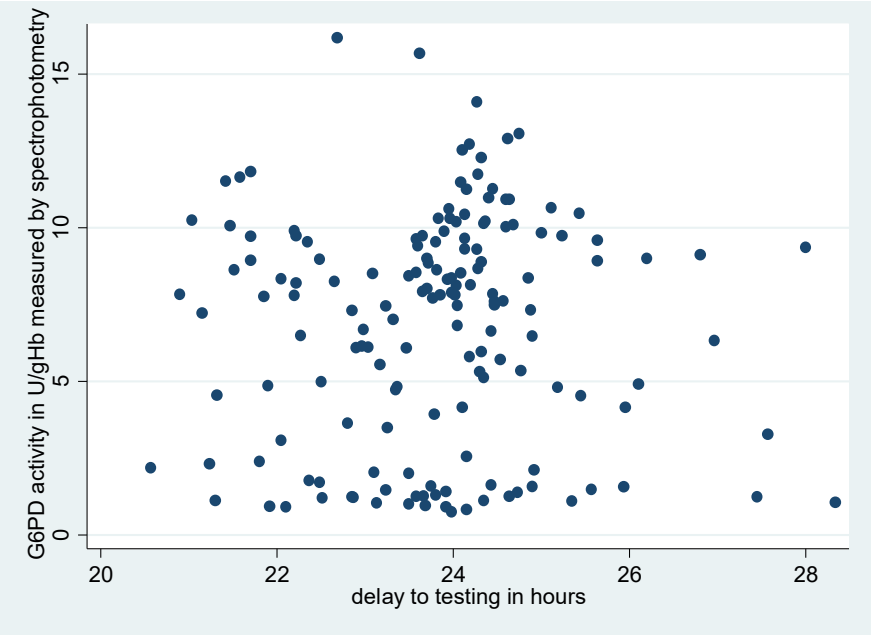

$r=-0.0110; p=0.890, n=158$

Supplement: S1 Fig — r = 0.0030; p = 0.970, n = 158. (PDF) [file pone.0206331.s001.pdf]

Supp. figure 7: Absolute fall in G6PD activity at 24 hrs and 13 days

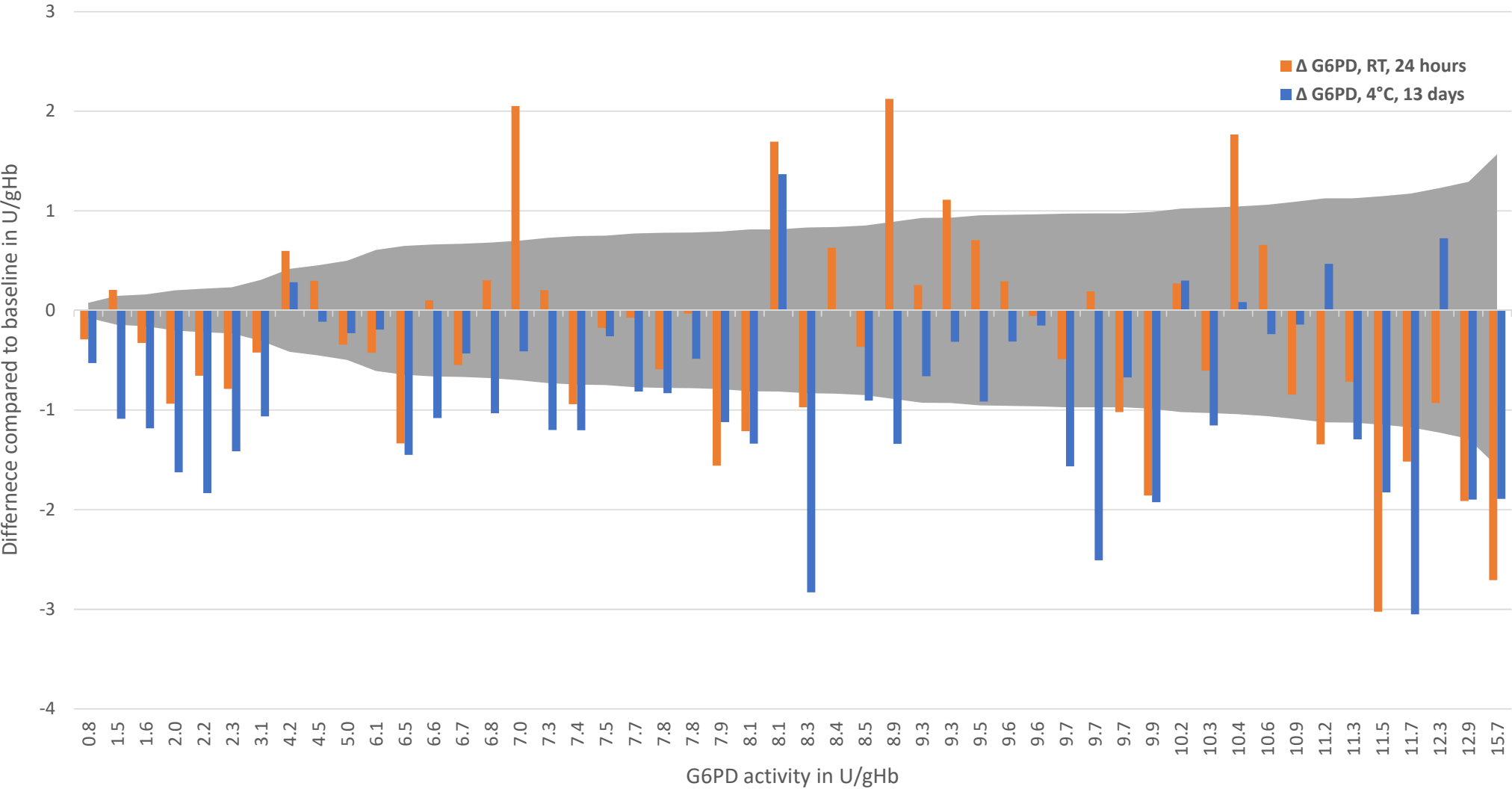

Supplement: S7 Fig — Grey shaded area indicates 10% of measurement, equivalent to max. variation of spectrophotometry. (PDF) [file pone.0206331.s007.pdf]
