# Supplementary material for: Field evaluation of quantitative point of care diagnostics to measure glucose-6-phosphate dehydrogenase activity
Source: PLoS One. 2018 Nov 2;13(11):e0206331. doi: 10.1371/journal.pone.0206331 (PMC6214512; doi:10.1371/journal.pone.0206331)
Supplement: S2 Fig — Repeatability of two measurements of the STANDARD G6PD TEST in the field- a) Scatter plot and b) Bland–Altman plot. a) rs = 0.9025; p<0.001, n = 106 b) Mean difference: - 0.66 U/gHb, 95% LoA: -2.04 to 3.36 U/gHb (grey shaded area). (PDF) [file pone.0206331.s002.pdf]

Supp. Figure 2: Repeatability of two measurements of the STANDARD™ G6PD in the field- a) Scatter plot and b) Bland – Altman plot

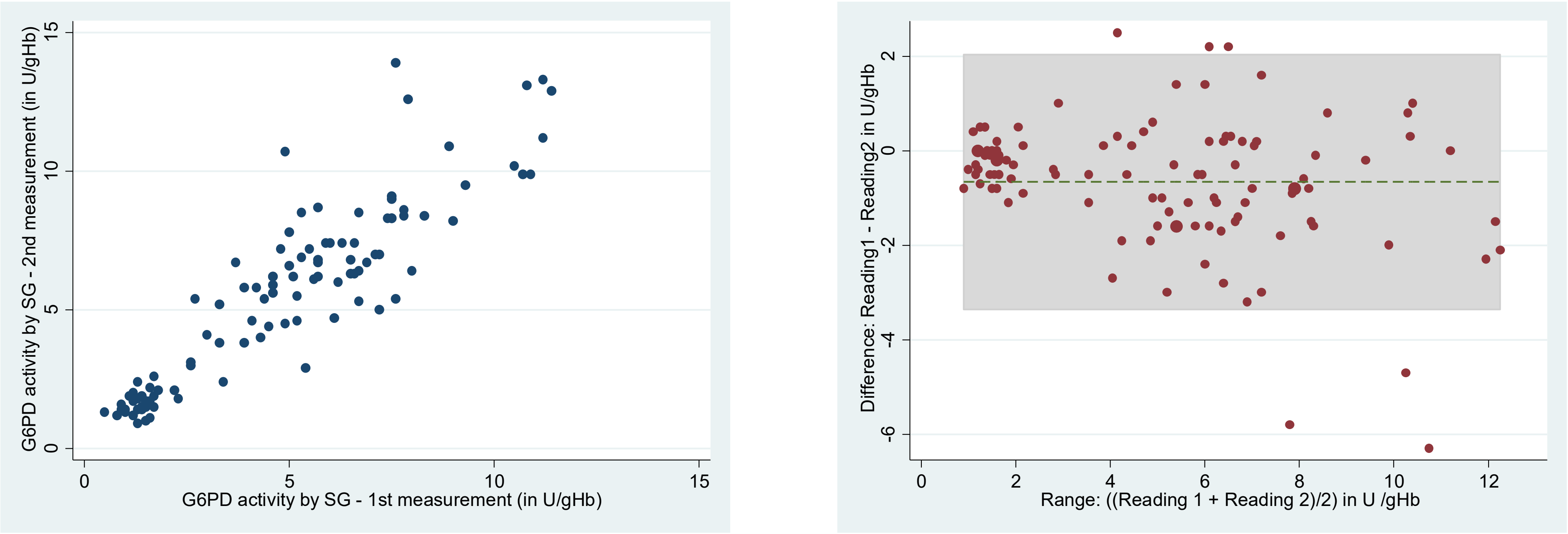

a) r_s=0.9025; p<0.001, n=106 b) Mean difference:- 0.66 U/gHb, 95% LoA: -2.04 to 3.36 U/gHb (grey shaded area)
