# Supplementary material for: Field evaluation of quantitative point of care diagnostics to measure glucose-6-phosphate dehydrogenase activity
Source: PLoS One. 2018 Nov 2;13(11):e0206331. doi: 10.1371/journal.pone.0206331 (PMC6214512; doi:10.1371/journal.pone.0206331)
Supplement: S3 Fig — Comparison of the STANDARD G6PD Test (SG) in the lab and field a) Scatter plot and b) Bland-Altman plot. a) rs = 0.8765; p<0.001, n = 106 b) Mean difference: -0.33 U/gHb, 95% LoA: -3.29 to 2.63 U/gHb (grey shaded area). (PDF) [file pone.0206331.s003.pdf]

Supp. Figure 3: Comparison of the G6PD STANDARD (SG) in the lab and field a) Scatter plot and b) Bland-Altman plot

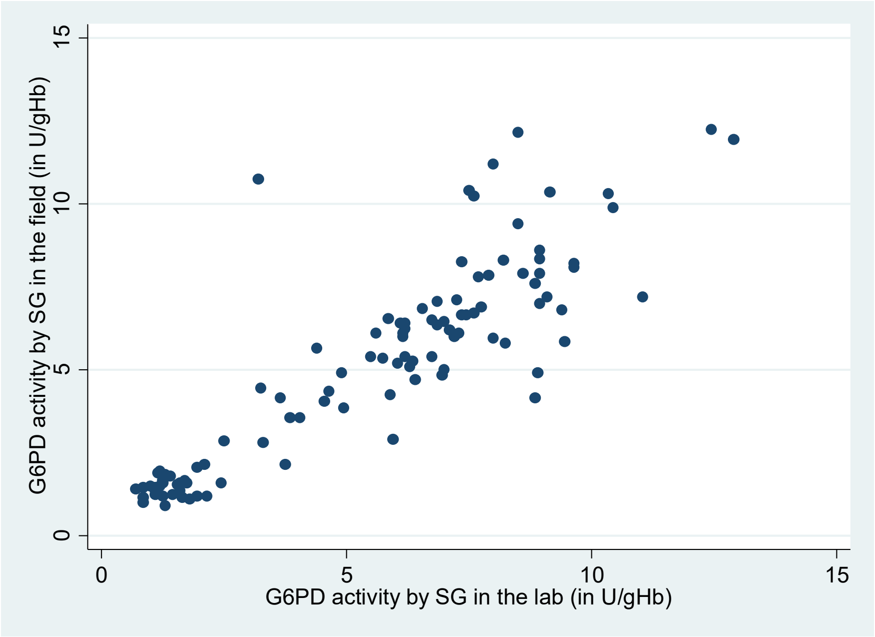

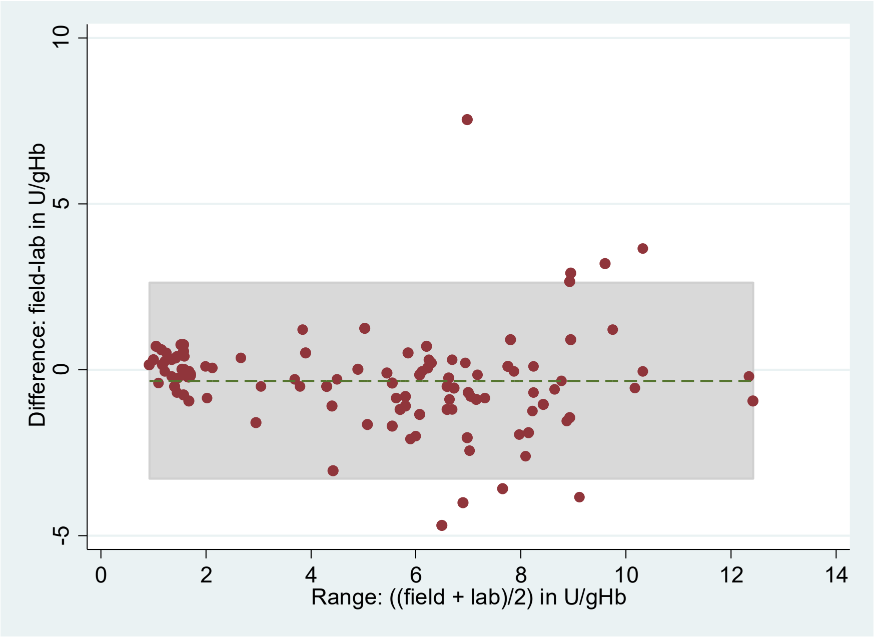

a) r_s=0.8765; p<0.001, n=106 b) Mean difference: -0.33 U/gHb, 95% LoA: -3.29 to 2.63 U/gHb (grey shaded area)
