# Supplementary material for: Field evaluation of quantitative point of care diagnostics to measure glucose-6-phosphate dehydrogenase activity
Source: PLoS One. 2018 Nov 2;13(11):e0206331. doi: 10.1371/journal.pone.0206331 (PMC6214512; doi:10.1371/journal.pone.0206331)
Supplement: S4 Fig — Comparison of the STANDARD G6PD Test (SG) in the field against spectrophotometry a) Scatter plot and b) Bland-Altman plot. a) rs = 0.9122; p<0.001, n = 106 b) Mean difference: -1.18 U/gHb, 95% LoA: -4.20 to 1.84 U/gHb (grey shaded area). (PDF) [file pone.0206331.s004.pdf]

Supp. Figure 4: Comparison of the STANDARD™ G6PD Test (SG) in the field against spectrophotometry a) Scatter plot and b) Bland-Altman plot

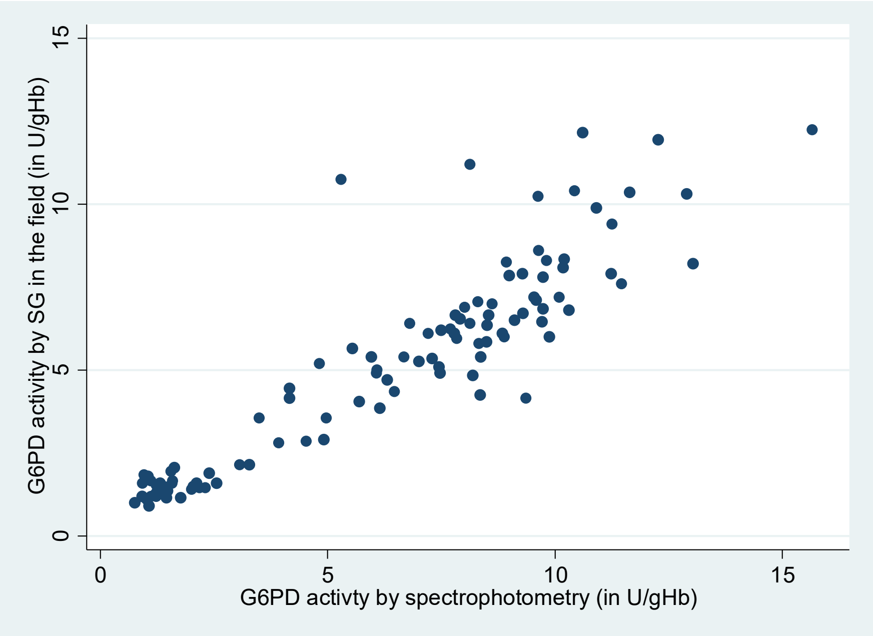

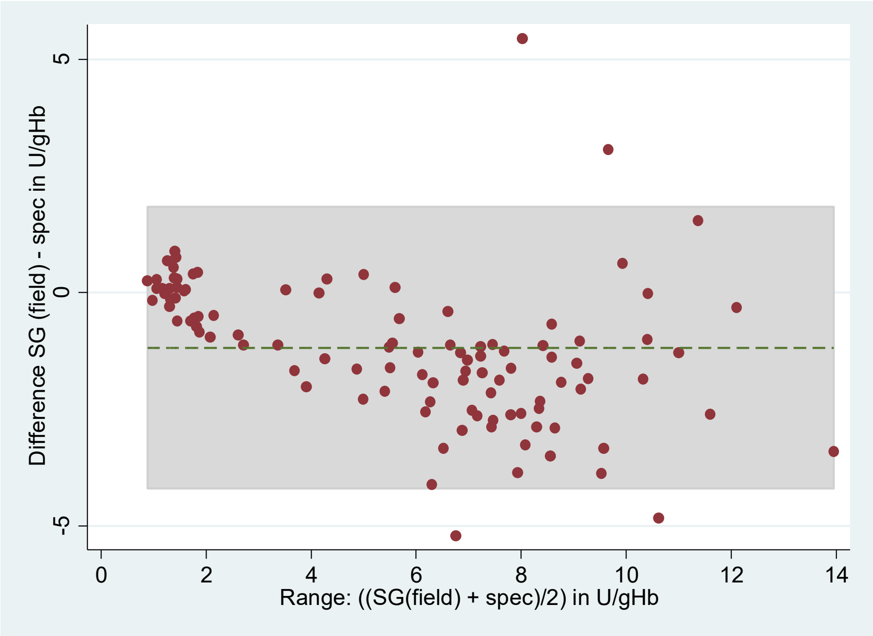

a) r_s=0.9122; p<0.001, n=106 b) Mean difference: -1.18 U/gHb, 95% LoA: -4.20 to 1.84 U/gHb (grey shaded area)
