# Supplementary material for: Field evaluation of quantitative point of care diagnostics to measure glucose-6-phosphate dehydrogenase activity
Source: PLoS One. 2018 Nov 2;13(11):e0206331. doi: 10.1371/journal.pone.0206331 (PMC6214512; doi:10.1371/journal.pone.0206331)
Supplement: S6 Fig — Comparison of the SG-Hb in the lab against CBC-HB a) Scatter plot and b) Bland-Altman plot. a) rs = 0.8892; p<0.001, n = 108 b) Mean difference: 0.37 g/dL, 95% LoA: -0.80 to 1.54 g/dL (grey shaded area). (PDF) [file pone.0206331.s006.pdf]

Supp. Figure 6: Comparison of the SG-Hb in the lab against CBC-HB a) Scatter plot and b) Bland-Altman plot

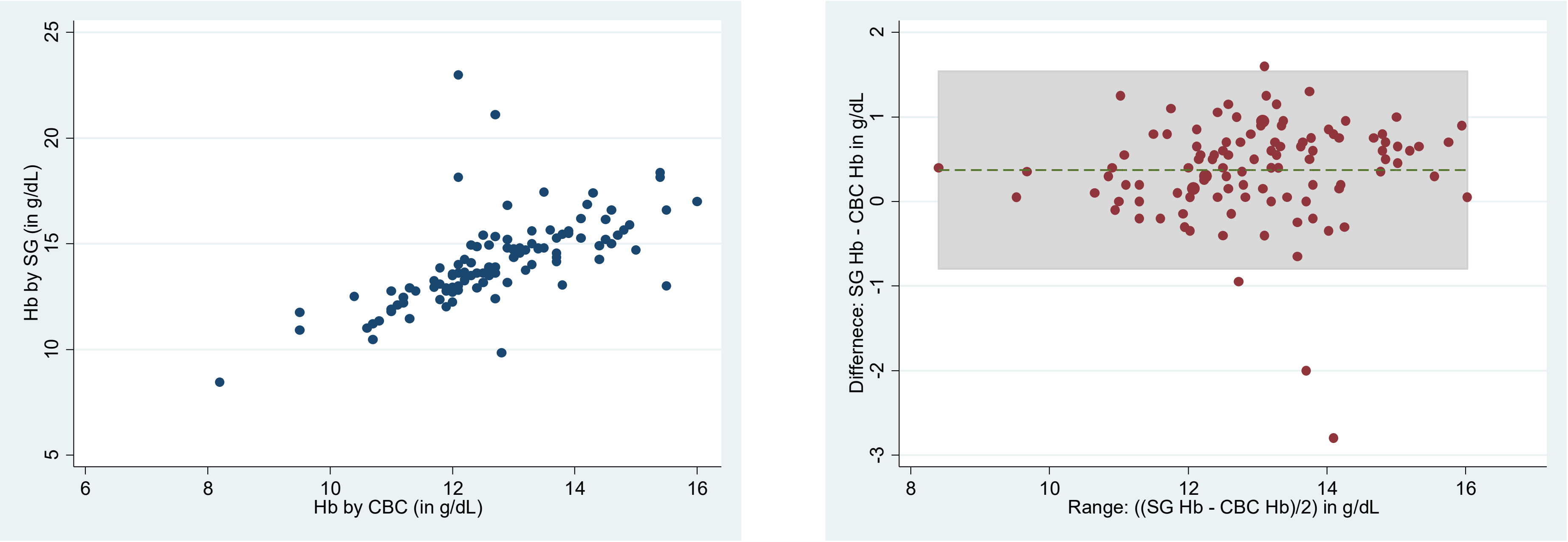

a) r_s=0.8892; p<0.001, n=108 b) Mean difference: 0.37 g/dL, 95% LoA: -0.80 to 1.54 g/dL (grey shaded area)
